# Increased longitudinal growth in rats on a silicon-depleted diet^☆^

**DOI:** 10.1016/j.bone.2008.04.014

**Published:** 2008-09

**Authors:** Ravin Jugdaohsingh, Mario R. Calomme, Karen Robinson, Forrest Nielsen, Simon H.C. Anderson, Patrick D'Haese, Piet Geusens, Nigel Loveridge, Richard P.H. Thompson, Jonathan J. Powell

**Affiliations:** a MRC Human Nutrition Research, Elsie Widdowson Laboratory, Fulbourn Road, Cambridge CB1 9NL, UK; b Nutritional Science Division, King's College London, School of Biomedical and Health Sciences, The Rayne Institute, St Thomas' Hospital, London SE1 7EH, UK; c Faculty of Pharmaceutical Sciences, University of Antwerp, Universiteitsplein 1, B-2610 Wilrijk—Antwerp, Belgium; d Biological Services Unit, King's College London, The Rayne Institute, St Thomas' Hospital, London SE1 7EH, UK; e USDA Grand Forks Human Nutrition Research Center, 2420 2nd Ave. N, Grand Forks, ND 58202, USA; f Experimental Clinical Pathology and Toxicology, Faculty of Medicine, University of Antwerp, Universiteitsplein 1, B-2610 Wilrijk—Antwerp, Belgium; g Biomedical Research Institute, University Hasselt, Campus Diepenbeek Agoralaan, B-3590 Diepenbeek, Belgium; h Department of Rheumatology, University Hospital, P. Debyelaan 25, 6229 HX Maastricht, The Netherlands; i Bone Metabolism Unit, Department of Medicine, University of Cambridge, Addenbrooke's Hospital (Box 157), Cambridge, UK

**Keywords:** Bone growth, Sprague–Dawley rats, Silicon deficiency, Orthosilicic acid supplementation, Bone and soft-tissue silicon concentrations, Tibia calcium and phosphorus

## Abstract

Silicon-deficiency studies in growing animals in the early 1970s reported stunted growth and profound defects in bone and other connective tissues. However, more recent attempts to replicate these findings have found mild alterations in bone metabolism without any adverse health effects. Thus the biological role of silicon remains unknown. Using a specifically formulated silicon-depleted diet and modern methods for silicon analysis and assessment of skeletal development, we undertook, through international collaboration between silicon researchers, an extensive study of long-term silicon depletion on skeletal development in an animal. 21-day old female Sprague–Dawley rats (*n* = 20) were fed a silicon-depleted diet (3.2 µg Si/g feed) for 26 weeks and their growth and skeletal development were compared with identical rats (*n* = 10) on the same diet but with silicon added as Si(OH)_4_ to their drinking water (53.2 µg Si/g water); total silicon intakes were 24 times different. A third group of rats, receiving a standard rodent stock feed (322 µg Si/g feed) and tap water (5 µg Si/g water), served as a reference group for optimal growth. A series of anthropometric and bone quality measures were undertaken during and following the study. Fasting serum silicon concentrations and especially urinary silicon excretion were significantly lower in the silicon-deprived group compared to the supplemented group (*P* = 0.03 and 0.004, respectively). Tibia and soft-tissue silicon contents did not differ between the two groups, but tibia silicon levels were significantly lower compared to the reference group (*P* < 0.0001). Outward adverse health effects were not observed in the silicon-deprived group. However, body lengths from week 18 onwards (*P* < 0.05) and bone lengths at necropsy (*P* ≤ 0.002) were *longer* in this group. Moreover, these measures correlated *inversely* with serum silicon concentrations (*P* ≤ 0.02). A reduction in bone growth plate thickness and an apparent increase in chondrocyte density were also observed in the silicon-deprived animals. No other differences were observed between the two groups, except for tibia phosphorus concentrations, which were lower in the silicon-deprived animals (*P* = 0.0003). Thus in this study we were unable to reproduce the profound deficiency state reported in rats and chicks in the early 1970s. Indeed, although silicon intake and circulating fasting serum levels differed between the silicon-deprived and silicon-supplemented animals, tibia and soft-tissue levels did not and may explain the lack of difference in bone quality and bone markers (except serum CTx) between these two groups. Markedly higher tibia silicon levels in the reference group and nutritional differences between the formulated low-Si and reference diets suggest that one or more co-factors may be absent from the low-Si diet that affect silicon incorporation into bone. However, evidence for urinary silicon conservation (to maintain tissue levels), changes in bone/body lengths, bone calcium:phosphorus ratio and differences at the growth plate with silicon deprivation are all novel and deserve further study. These results suggest that rats actively maintain body silicon levels via urinary conservation, but the low circulating serum silicon levels during silicon deficiency result in inhibition of growth plate closure and increased longitudinal growth. Silicon-responsive genes and Si transporters are being investigated in the kidneys of these rats.

## Introduction

Silicon (Si) is the second most abundant element in the Earth's crust [1] and is found in all living organisms including man, where the highest concentrations are reported in bone and other connective tissues [2]. It is present in blood at concentrations that are in the range of typical physiologically important elements, such as copper and zinc [3]. It is a major trace element in animal diets, and humans ingest between 20–50 mg/d in the Western diet [4–6]. Main dietary sources are whole grain cereals and their products (including beer), rice, some fruits and vegetables and drinking water, especially bottled mineral waters [4,6,7]. Meat and dairy products have low levels of Si [6,7]. Intestinal absorption is dependent on the chemical form: i.e. monomeric silicic acid (orthosilicic acid), present in beverages such as beer and water, or partially produced by digestion of foods in the gut lumen, is efficiently absorbed whereas polymeric (colloidal) silica are poorly absorbed [4,8]. Thus it has been suggested that Si may have an important role in biology beyond being a ‘ubiquitous contaminant’ [9].

Indeed, Si-deprivation experiments in the early 1970s in growing chicks [10] and rats [11] reported that Si was required for normal growth and development in higher animals, since Si deficiency produced stunted growth and profound defects in bone and connective tissues [10,11]. Skull deformities (shorter skull and distortion around the eye sockets), shorter, thinner and more flexible limbs bones that were easily fractured and, the absent of wattle and comb (in the chick) were reported in the Si-deprived animals [10,11]. In the rat, hair loss, seborrhoea, loss of muscle tone, disturbances in enamel development and impairment of incisor pigmentation were also observed [11]. These findings, indicative of a role for Si in connective tissue formation, have been supported by further observations such as: (i) the presence of high concentrations of non-dialysable Si in connective tissues and their components ([12] and Jugdaohsingh et al., unpublished data), (ii) the localisation of Si at the growing front of bone [2], (iii) *in vitro* studies showing increased synthesis of matrix polysaccharides and proteins with Si supplementation [2,13] and (iv) the requirement of Si for optimal prolyl hydroxylase activity, the enzyme involved in collagen synthesis [2,13].

More recently, a number of *in vitro* studies using human osteoblast-like cells, and *in vivo* studies in animals and man, have also added to the evidence that Si may be beneficial for bone formation, and for bone and connective tissue health [2,14–25]. The exact mechanisms of action of Si on bone are still unclear, and both a metabolic role (a co-factor for prolyl hydroxylase) and a structural role (stabilisation of collagen) have been proposed [2,13,25]. Thus, whether Si is truly required for optimal skeletal growth in humans or other animals is not known.

More recent studies investigating the essentiality of Si in growing animals 26–35] have failed to reproduce the dramatic results reported by Carlisle [10] and by Schwarz and Milne [11], although various moderate effects on bone metabolism have been observed. Differences in experimental conditions such as duration of the experiment, age and gender of the animals, Si source and route of administration, as well as overall nutritional compositions of the basal diet may all contribute to the inter-experimental variance. Notably, early studies used simplified amino acid-based diets with, apparently, the addition of sufficient and balanced amounts of vitamins (at 1%) and other minerals for optimal/near optimal growth [36,37]. However, a co-deficiency cannot be ruled out. The exact composition of the rat basal diet was not found on literature search, but the composition of basal diet used in the chick studies was similar to the formulated low-Si diet used here (i.e. all vitamins and minerals were present; see Table 1), apart from the chick diet being amino acid-based rather than casein/corn-based used here [37]. Moreover, it is unlikely with the methodology of the time that the absolute content of Si in the basal diet could be assessed, although in the rat study it was reported as < 5 µg Si/g feed [11].

Since then only one group [29–34] has been able to achieve similar low dietary levels of Si for animal studies (*circa* 3 µg Si/g feed), while methods for accurate analysis of Si and other micronutrients, as well as measurement of skeletal health, have greatly advanced. Thus, drawing on the collaborative expertise of some research groups in Si and health we set out to determine the role of Si in growth and development, and in particular skeletal development, in the rat using a specifically formulated low Si-containing feed and a low Si-containing drinking water with and without Si supplementation. A group of rats on a standard rodent stock feed and drinking water served as reference for normal anthropogenic measures. The standard rodent stock feed was higher in Si content than our formulated low-Si feed. Still, however, due to other nutritional differences, the two diets were not compared in this study with regards to the effect of silicon.

## Materials and methods

### Experimental groups

Forty weanling (21 d old) female Sprague–Dawley rats (B&K Universal Ltd; Hull, UK) were separated into eight plastic cages with stainless-steel-grid bases (North Kent Plastics, Rochester, UK) with five rats per cage. Female rats were chosen, as they are generally less aggressive and more easily handled than males. This study was started prior to our 2004 publication reporting a more marked association between dietary Si intake and bone mineral density in pre-menopausal women compared to men and post-menopausal women, suggesting a Si–estrogen interaction [18]. After acclimatisation for three days on a standard pelleted rodent feed (B&K Rat and Mouse Standard Diet; B&K Universal Ltd) and drinking water (tap water), rats were divided into three dietary groups.

#### Group 1 (Si-deprived)

This group consisted of twenty rats (see below). They were weighed on a precision balance accurate to 0.1 g (Sartorius Ltd, Epsom, UK) and their length, including tail, measured with a ruler to the nearest 0.1 cm. Rats were then fed *ad libitum* a specifically formulated non-pelleted low-Si rodent feed (prepared by one of the authors (FN) as previously described [29–34]) containing all essential nutrients, but with a Si content of only 3.2 ± 0.6 μg Si/g feed (*n* = 6 batches; see Table 1). The vitamin K content was 15-fold lower compared to standard rodent stock feed, but was identical to the vitamin K requirement published for laboratory animals [38]. These animals also received ultra-high purity (UHP) water (18 MΩ cm^− 1^, from an Elga water purifier; High Wycombe, UK), of low Si content (15.2 ± 9.6 ng Si/g; *n* = 5), *ad libitum*. The rat feed was easily accessible from a stainless-steel food tray and replaced with fresh food every 2–3 days. Similarly, drinking water (UHP water) in the plastic bottles (North Kent Plastic) was renewed every other day. Stainless steel grid base cages were used to minimise coprophagia.

This group consisted of twice the number of animals as the Si-supplemented group as it was planned that half the animals would be supplemented with Si (in their drinking water) on observation of external signs of Si deficiency, to show that it was the effect of Si-depletion and not some other factor.

#### Group 2 (Si-supplemented)

This group consisted of ten rats that were similarly treated and maintained as described above (Group 1), but the UHP drinking water was supplemented with silicon at 53.2 ± 0.6 μg Si/g (*n* > 10), in the form of readily absorbable orthosilicic acid (see below). This is the highest concentration that can be prepared in solution while maintaining silicon in the soluble, monomeric and highly bioavailable form [8,39].

#### Group 3 (reference group)

This group consisted of ten rats housed and measured as described above (Group 1), but maintained on standard rodent stock feed (B&K Rat and Mouse Standard feed containing 322 ± 47 µg Si/g feed, *n* = 5 batches; see Table 1) and tap water (5.04 ± 1.12 μg Si/g; *n* = 5), both *ad libitum*. This was the reference group for normal anthropometric and bone quality measures.

### Study design

Rats were maintained as described above (at 22 °C, with 12/12 h light/dark cycle) with free access to feed and water for 26 weeks. A sample of the feeds and drinking waters was collected every month and analysed for total Si content (see below). Feed and water intake was measured per cage (5 animals to a cage) at 17 and 25 weeks. Animals were not pair-fed in this study, as intakes were not expected to be different between the groups. Previous studies reported that Si supplementation does not impact on feed consumption, body weight gain, or feed efficiency [40].

Rat weights and lengths were measured weekly. A blood sample from the tail vein was collected monthly from six-hour fasted rats (only access to UHP water was allowed) in each group for longitudinal analysis of bone markers (CTx-I, osteocalcin, IGF-1, CTx-II; see below). A urine sample was also collected towards the end of the study (week 25) from fasted rats for assessment of urinary bone resorption markers and urinary Si excretion. Prior to sacrifice, rats were double-labelled with tetracycline-HCl for bone histomorphometric analyses (see below).

After 26 weeks all animals were sacrificed, equally between groups, over a period of eight days to allow time for tissue processing. Animals were starved from 1700 h the day before by removal of their feed and drinking water, and only UHP water of low Si content was allowed. Rats were sacrificed by asphyxiation (schedule 1 method) with carbon dioxide gas in a specially constructed chamber. Animals were then weighed and the peritoneum opened longitudinally. The abdominal aorta and vena cava, mesenteric arteries and heart were drained using a 19-gauge needle (Terumno; Terumno Europe NV) and 10 mL low Si-contaminating syringe (Monovette; Sarstedt, Beaumont Leys, UK). After clotting, blood samples were centrifuged (1500 rpm for 10 min at room temperature), and serum separated into transport tubes (Medfor Products Ltd; Farnborough, UK) and stored at − 70 °C for later analysis of bone markers and Si concentration (see below). Kidneys, spleen and liver were removed, washed thoroughly with UHP water, blotted dry on non-shedding paper towels and placed in 25-mL pre-weighed universal polypropylene tubes (Sarstedt) and their weight determined to the nearest 0.1 mg before frozen storage (− 20 °C, livers at − 80 °C) for later microwave-assisted acid digestion and analysis of Si content (see below). Femurs, tibias, humeri, and lumbar vertebrae (between first rib and pelvis) were dissected and freed of soft tissue (i.e. cleaned to the bone). The right tibia was immediately placed into Burkhardt fixative solution (see below) for 24 h at 4 °C and thereafter stored in 70% ethanol for later histomorphometric analyses (see below). For the other dissected bones, their mass (to nearest 0.1 mg) and length (to nearest 0.1 mm using callipers; VWR International) were determined before being placed into tubes and stored at − 20 °C. To avoid dehydration and loss of moisture during storage, dissected bones were stored with 1–2 drops of UHP water. Dissected bones were analysed for bone mineral density (BMD), Si and other bone-associated mineral content, hydroxyproline concentration, mechanical strength and histomorphometry (see below).

The study was approved by King's College London Animal Ethics Committee and by the UK Home Office (Animals Scientific Procedures Act 1986; Scientific Procedures on Living Animals).

### Formulated low-Si rat feed

The specifically formulated rodent feed was prepared/mixed fresh every month (see Table 1 for list of constituents) in the US and shipped to the UK where the experiment was undertaken and where it was stored at − 20 °C and thawed and used as required.

### Preparation of orthosilicic acid

Orthosilicic acid solution (1.9 mmol/L Si), for the Si-supplemented rats, was prepared fresh, weekly, as described previously [8,39,41]. Briefly, concentrated basic sodium silicate (Aldrich Chemical Co., Poole, UK) was diluted in 2.5 L ultra-high purity water, followed by pH neutralisation to 7.2 with 5 mol/L hydrochloric acid (volumetric standard; Aldrich Chemical Co.). The solution was allowed to stand at room temperature for at least 24 h prior to use. A 10 mL aliquot was taken for total Si analysis.

### Silicon intake

Feed and water intakes were measured towards the end of the study. Briefly, at 17 and 25 weeks the feed tray and water bottles of each cage were appropriately filled and weighed (to the nearest 0.1 g). Twenty-four hours later the feed tray and water bottles were re-weighed to measure the loss in mass due to intake by the rats. This 24 h measurement was immediately repeated for another three subsequent 24 h periods and the mean feed and water intakes were thus calculated for each cage and each group and thus also mean 24 h (daily) Si intake for each group.

### Monthly blood collection and preparation

A fasting blood sample was collected monthly from half the animals in each group (10 rats in the Si-deprived group; five from each of the other two groups), but alternating monthly between the two halves in each group. Animals were fasted from 0900 h by removal of their feed but all had access to UHP water. Six hours later, at 1500 h, up to a maximum of 1 mL blood/rat was collected by venepuncture of the tail vein using a 23-gauge needle (Terumo Europe NV, Belgium) but without use of a syringe. Prior to blood collection, rats were ‘warmed’ using a heating pad and box (Tecniplast, Kettering, UK) and then restrained (Vet-Tech Solutions Ltd, Congleton, UK). Blood was collected directly into 1.5 mL Eppendorf tubes (Greiner Bio-One Ltd, Stonehouse, UK) and allowed to clot before centrifugation at 3000 rpm for 10 min at room temperature. Serum was separated into Eppendorf tubes and stored at − 70 °C.

### Fasting urine sample

Three weeks prior to sacrifice, a spot urine sample was collected by bladder massage from 6-h fasted rats. Urine samples were successfully collected from eight rats in the Si-deprived group, five in the Si-supplemented group and seven in the standard rodent stock feed-fed reference group and were stored in Eppendorf tubes at − 70 °C until later analysis of Si concentrations and bone resorption markers.

### Tetracycline double labelling

To locate the growing bone front and measure the rate of bone apposition/formation over a six-day period, and the influence of dietary Si, rats were injected with tetracycline 7 days and 1 day prior to sacrifice (i.e. on two occasions, six days apart). Tetracycline localises to the growing bone front, the site of bone formation/mineralisation. Tetracycline-HCl (Sigma Chemical Co) solution, 10 mg/mL, was prepared just prior to use by dissolving the powder in sterile saline (0.9% w/v NaCl) and the solution sterilised by filtration through a 0.2 μm membrane filter (Nalgene, surfactant free cellulose acetate; VWR International). Animals were administered tetracycline (30 mg/kg body weight) by injection into the intraperitoneal cavity (lateral below the navel) using a 25-gauge needle, 7 days and 1 day prior to sacrifice.

### Burkhardt fixative solution

The right tibia from each rat, collected at sacrifice, was immediately fixed in Burkhardt fixative [42], prior to embedding in methyl methacrylate and sectioning for histomorphometric analyses. Burkhardt fixative fixes the tissue components in place and reduces leaching of the tetracycline labels. Burkhardt solution was prepared fresh, daily, by mixing 12.5 mL neutralised formaldehyde (37% w/v) with 24 mL absolute methanol, 0.5 mL phosphate buffer (prepared by dissolving 1.92 g disodium hydrogen phosphate and 0.348 g potassium dihydrogen phosphate in 100 mL UHP water), and 0.5 mL 6.16% glucose (prepared by dissolving 6.16 g glucose monohydrate in 100 mL UHP water). The solution was cooled to 4 °C prior to use.

## Analyses

### Bone mineral density

The right femur and lumbar vertebrae (L1–L4) were analysed for bone mineral content (BMC) and bone mineral density (BMD) by dual energy X-ray absorptiometry (DEXA; Hologic QDR-2000; S/N 2484) adapted for use with small animals. Measurements at the femur were at four regions of interest (from the midshaft (H1; primary cortical bone) to the distal metaphysis (H4; trabecular bone)) and from the total femur as previously described [15]. Similarly, for the lumbar spine, measurement was at the individual vertebra (L1, L2, L3 and L4) as well as for the total lumbar spine (L1–L4). Values are the average of two measurements.

### Bone markers

#### Serum

Fasted serum samples, collected at sacrifice, were analysed for a number of bone formation and resorption markers using commercially available kits. The bone formation markers were osteocalcin (Rat-Mid Osteocalcin; Nordic Bioscience Diagnostics A/S, Denmark) and alkaline phosphatase (Sigma Chemical Co.). The resorption markers were C-terminal telopeptide of type I collagen (CTx-I or RatLaps; Nordic Bioscience Diagnostics A/S), intact parathyroid hormone (Immunotopics Inc Rat intact PTH; Quidel Diagnostics, Wheatley, UK), tartrate resistance acid phosphatases (SBA Sciences RatTRAPS; Quidel Diagnostics) and free (i.e. non-protein bound) and total pyridinium (Metra PYD; Quidel Diagnostics) and deoxy-pyridinium (Metra DPD; Quidel Diagnostics) cross-links. Osteopontin (TiterZyme EIA; Assay Designs Inc, Ann Arbor, USA) and sialic acid (Roche Diagnostics Ltd, Lewes, UK) were also measured in these samples. For the serum samples collected monthly during the study, CTx and osteocalcin were measured (*a priori*) because of the likely role of Si in collagen turnover (CTx) and bone formation (osteocalcin). Insulin-like growth factor 1 (IGF-1; Octeia Rat/Mouse IGF-1; IDS Ltd, UK), total serum estradiol (Department of Chemical Pathology, St Thomas' Hospital) and serum CartiLaps (CTx-II; Nordic Bioscience Diagnostics A/S) were also measured *post hoc* (i.e. after assessment of the other results) in these monthly serum samples.

#### Urine

Fasting urine samples were analysed for bone resorption markers; CTx-I (RatLaps; Nordic Bioscience Diagnostics A/S), helical peptide (Metra, Quidel Diagnostics) and free and total PYD and DPD (Metra, Quidel Diagnostics). Concentrations of these markers in urine were corrected/normalised for urinary creatinine (Metra, Quidel Diagnostics).

#### Left femur

The left femur was analysed for hydroxyproline as a measure of its collagen/matrix content. The procedure was based on acid hydrolysis of the bone and subsequent determination of the free hydroxyproline in the hydrolysates [33,43]. Briefly, chloramine-T hydrolyses hydroxyproline to a pyrrole derivative. This was then reacted with 4-dimethylaminobenzaldehyde (p-DAB) in an acid medium to form a chromophore (Ehrlich's reaction) the absorption of which was measured at 570 nm. The colour (chromophore) was enhanced by the addition of isopropanol.

### Bone mechanical properties

Torsional properties of the left femur were measured as described by Jiang et al. [44]. The test machine (Biomechanical Laboratory, Cleveland, OH, US) allowed high strain rates (loading time to fracture less than 100 ms) which are representative of *in vivo* conditions. Data from strength and displacement transducers were recorded with a Biomation Waveform Recorder model 1015 and processed with a PC. Applied load as a function of time was recorded continuously. The femur was embedded at both ends in rectangular epoxy blocks, fitting the grips of the torsional test machine. After the torsion test, no permanent distortion between the bone and the embedding material could be observed.

### Bone histomorphometry

Histomorphometric analyses were performed as previously described [45]. Measurements were conducted at the primary spongiosa.

### Elemental analyses

#### Microwave-assisted acid digestion

All feeds and tissues (right kidney, spleen, liver and left tibia) were digested in acid prior to elemental analysis. Briefly, feeds (0.1–0.3 g) were digested in 10 mL of a 1 + 1 mixture of UHP nitric acid (65% w/v; Fluka; Aldrich-Sigma Chemical Co.) and UHP water in acid-cleaned 100 mL TFM vessels in an Ethos Plus Microwave Labsystem (Milestone Srl; Sorisole, Italy). Microwave conditions were: 10 min ramp to 180 °C and maintained at 180 °C for 15 min. Blanks (acid mixture without sample) were also prepared and ‘digested’ in parallel. The cooled digested samples and blanks were transferred into cleaned, pre-weighed polypropylene 60 mL bottles (WVR International Ltd) and diluted with 10 mL UHP water.

The whole right kidney (0.9–1.5 g), spleen (0.4–1.1 g), liver (~ 0.75 g) and left tibia (0.5–0.7 g) were digested under the same conditions as described above, but in 10 mL, 30 mL, 16 mL and 10 mL, respectively, of 1 + 3 mixture of UHP nitric acid and UHP water. Digested samples and blanks were analysed for Si and other elements/minerals as described below (ICPOES analysis).

#### ICPOES analysis

Total elemental analysis was carried out by inductively coupled plasma optical emission spectrometry (ICPOES; Jobin-Yvon JY24; Instrument S.A., Longjumeau, France) with a V-groove nebuliser and Scott-type double pass spray chamber and a sample flow rate of 1 mL/min. Peak profiles were used as before [46,47], using a window size of 0.1 nm (0.05 nm either side of the peak) with 54 increments per profile. Analytical lines used were 251.611 nm (Si), 396.847 and 315.887 nm (Ca), 279.553 and 293.654 nm (Mg), 259.940 nm (Fe), 213.856 nm (Zn), 324.754 nm (Cu), 257.610 nm (Mn), 330.298 nm (Na), 766.490 nm (K), 249.678 and 249.773 nm (B) and, 206.149 and 267.716 nm (Cr). Phosphorus was analysed by ICPOES using a Leeman DRE ICP (Leeman Labs Inc., Hudson, USA) at 214.91 nm as this instrument was superior for near-UV analyses. Serum P was analysed with a JY2000 (Jobin-Yvon). Samples were analysed with sample-based standards prepared by diluting aliquots of the stock element standard solutions (100 or 1000 mg/L; Merck Ltd; Sigma-Aldrich Chemical Co.; or Leeman Labs Inc.) in pools of the different sample types. Samples from all the animals were analysed in a single batch.

#### Serum samples

Serum samples (1 mL) collected at sacrifice were diluted with 4 mL 0.26% (v/v) UHP nitric acid and analysed for Si and Mn with an integration time of 1 s per increment. Samples were further diluted (1 +9 with 0.26% nitric acid) for analysis of the other elements (Ca, Mg, Zn, Cu and K); with an integration time of 0.5 s per increment. Serum P was analysed by acid digestion of 1 ml of the 1 + 4 diluted serum with equal volume of 1:1 mixture of concentrated UHP nitric acid and hydrogen peroxide and then dilution with 3 ml of UHP water.

#### Urine samples

Fasting spot urine samples were diluted 1:10 with UHP water prior to analysis for Si with an integration time of 0.5 s per increment. Silicon concentrations were corrected for urinary creatinine.

#### Feed and drinking water

Digested samples of feeds and untreated drinking waters, collected monthly, were analysed for Si with an integration time of 0.5 s per increment. The feeds were also analysed for Ca, K, Mg, Mn, Zn, Fe, Cu, Cr and B to confirm the manufacturer's reported values.

#### Tissue samples

Digested tissues (right kidney, spleen, liver and left tibia,) were analysed for Si with an integration time of 0.5 s per increment. Copper, Zn, Ca, Mg, K, Na, Mn, Fe and P were also measured in the digested tibias without (for Cu and Mn) or with further dilution (1:10 for Zn, Fe, K and Na, and 1:1000 for Ca, Mg and P) of the samples with UHP water. Elements were measured with an integration time of 0.3–0.5 s per increment.

### Statistical analyses

Results are expressed as means ± SD, unless otherwise stated. Differences between the Si-deprived and Si-supplemented groups were analysed by unpaired two-tailed Student's *t*-test with significance taken as *P* < 0.05. Results are reported without adjustment for multiple comparisons, but we have also reported where results would remain significant with a simple and non-conservative Bonferroni correction (i.e. P/n) where results are part of a larger group (e.g. bone markers). Repeat measurements (RM)-ANOVA was used for assessment of weekly body weights and lengths and, monthly serum CTX and osteocalcin measurements over the study period. These were conducted in SPSS for Windows (version 11; SPSS Inc., Chicago, USA).

## Results

### Silicon intakes

Since there were no marked differences in intake of feed and water at 17 and 25 weeks, the mean intakes over the two periods are given for simplicity for each group. Thus the estimated mean ± SD daily intake of Si at 17 and 25 weeks (i.e. from feed plus water) was 0.17 ±0.04 mg/kg body weight for the rats in the Si-deprived group and 4.08 ± 0.74 mg/kg body wt in the supplemented group. Dietary silicon intake in the Si-supplemented animals was thus 24 times that of the Si-deprived group.

In the standard rodent stock feed-fed reference group, estimated daily Si intake was 18.51 ± 0.65 mg/kg body weight. Again, as previously stated, due to other nutritional differences (see Table 1), the two diets were not compared in this study with regards to the effect of silicon on the rats. Rats on the standard rodent stock feed served *only* as a reference for normal anthropogenic measures.

### Tissue Si measurements

**Fig. 1:**
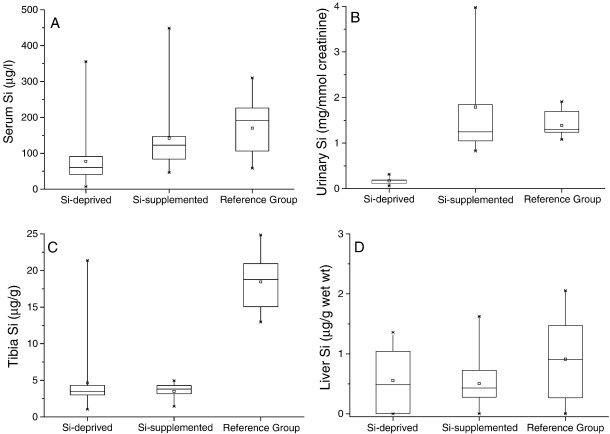
Fasting serum Si (A) and urinary Si excretion (B), and Si contents of the left tibia (C) and liver (D) of the rats in the Si-deprived group (*n* = 20 rats), Si-supplemented group (*n* = 10) and reference group (*n* = 10 rats on the standard rodent stock feed). [Urine samples were only available for eight rats in the Si-deprived group, five rats in the Si-supplemented group and seven rats in the reference group.] Fasting serum Si and urinary Si excretion were significantly lower in the Si-deprived group compared to the Si-supplemented group (*P* = 0.03 and *P* = 0.004, respectively; unpaired two-tailed Student's *t*-test). However, there were no significant differences in Si contents of the left tibia and the liver between the two groups.

Following 26 weeks of dietary intervention, we compared Si concentrations of the serum, urine, tibia and soft tissues (liver, kidney and spleen) between animals on the Si-deprived diet and those on the same, but Si-supplemented, diet (via their drinking water). Serum concentrations of Si (78 ± 77 µg Si/L in the Si-deprived vs. 142 ± 116 µg Si/L Si-supplemented group), and especially urinary concentrations of Si (0.171 ± 0.09 mg Si/mmol creatinine in the Si-deprived vs. 1.79 ±1.28 mg Si/mmol creatinine in the Si-supplemented group), differed significantly between the two groups (*P* = 0.029 and *P* = 0.0038, respectively; Figs. 1A and B). Tissue concentrations, however, were the same (Figs. 1C and D; liver is shown as representative for soft tissues; 0.55 ± 0.53 µg Si/g vs. 0.50 ± 0.47 µg Si/g in the Si-deprived vs. Si-supplemented group). We also undertook Si analyses of the tissues and bio-fluids from the reference group of rats ingesting standard rodent stock feed and tap water for the same period. Silicon concentrations in serum (170 ± 79 µg Si/L), urine (1.39 ± 0.31 mg Si/mmol creatinine) and liver (0.91 ± 0.73 µg Si/g) were similar between the reference and Si-supplemented groups, but Si concentrations in bone (tibia) were much higher in the reference group (18.4 ± 3.7 µg Si/g compared with 4.7 ± 4.7 µg Si/g and 3.5 ± 1.2 µg Si/g in the Si-deprived and Si-supplemented groups, respectively; *P* < 0.0001; Fig. 1C). There were some other marked nutritional differences including Si content (2–200-fold) between the standard rodent stock feed and the formulated low-Si diet (Table 1; e.g. vitamins K, B2, B3, and B5 and minerals including cobalt, magnesium, iron and zinc) that restricted our further comparisons to the Si-deprived versus the Si-supplemented groups, where the *only* difference was the addition of orthosilicic acid to the drinking water of the latter group. Data are however reported for the reference group of animals (see tables and figures).

### Bone measurements

**Fig. 2:**
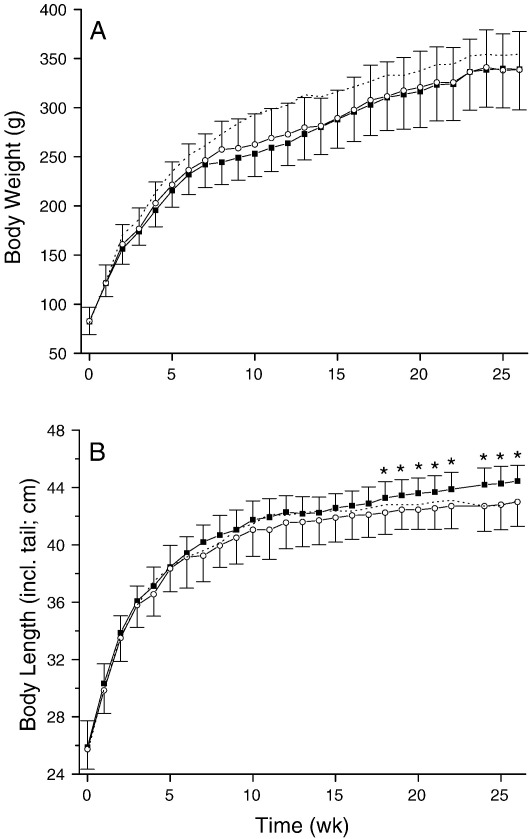
Body weights (A) and lengths (B) of the rats in the Si-deprived (solid squares; *n* = 20 rats) and Si-supplemented (open circles; *n* = 10 rats) groups. There was no significant difference in body weights between the two groups; however, rats in the Si-deprived group were significantly longer from weeks 18 onwards (*P* ≤ 0.046 → *P* < 0.01; RM-ANOVA) compared to the Si-supplemented group. Body weight and length are also shown for the standard rodent stock feed-fed group as a reference for normal rats (dotted lines).

**Fig. 3:**
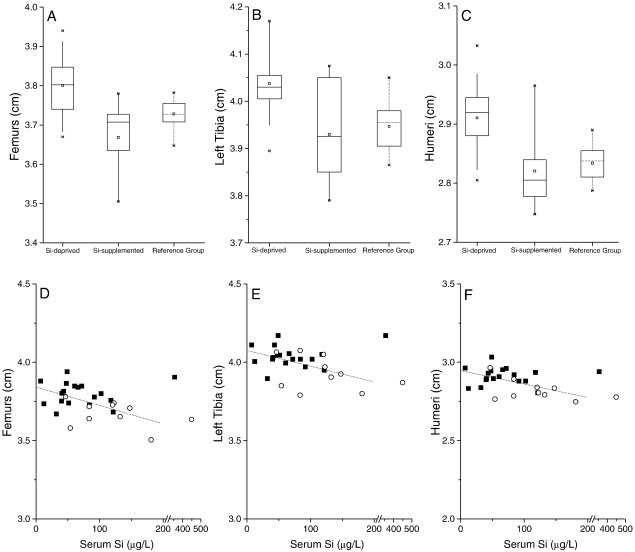
Length of femurs (A), left tibia (B) and humeri (C) of the rats in the Si-deprived (*n* = 20) and Si-supplemented (*n* = 10) groups and correlations between fasting serum Si concentrations at necropsy and lengths of, femurs (D), left tibia (E), and humeri (F), of rats in the Si-deprived (squares) and Si-supplemented (circles) groups. The limb bones of the rats in the Si-deprived group were significantly longer compared to the rats in the Si-supplemented group (*P* = 0.0002 at the femur; *P* = 0.002 at the left tibia; and *P* = 0.0006 at the humerus; unpaired two-tailed Student's *t*-test) and inversely correlated with fasting serum Si concentrations (*r* = − 0.53, *P* = 0.006 for femurs; *r* = − 0.47, *P* = 0.016 for the left tibia; and *r* = − 0.52, *P* = 0.007 for humeri). Two outliers (one in each group), with markedly higher Si concentrations (> 300 µg/L) compared to the group mean/median, are likely to represent inadvertent contamination upon sample collection (despite the great care taken) and are shown, but were not included in the correlations. Lengths of the limb bones are also shown for the standard rodent stock feed-fed group as a reference for normal rats.

**Fig. 4:**
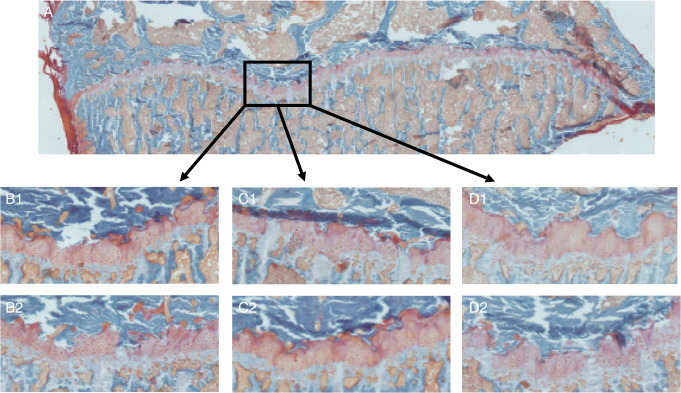
Growth plate of the right tibia (A) and higher magnification images in the Si-deprived (B1 and B2), Si-supplemented (C1 and C2) and standard rodent stock feed-fed (D1 and D2) groups. Growth plates from two animals (1 and 2) are shown as representative for each group. Growth plate thickness was reduced, while chondrocyte cell density appears to be slightly increased in the Si-deprived group (B1 and B2) compared to the Si-supplemented and standard rodent stock feed-fed reference groups.

Fasting serum concentrations of bone-associated elements (Ca, Mg, K, Zn, Cu and Mn) were not different between the two groups (Table 2). Serum P was lower in the Si-deprived group (242 ± 19 mg P/L vs. 295 ± 33 mg P/L in the Si-supplemented group) but this was not statistically significant (*P* = 0.134). Similarly, elemental tibia concentrations did not differ (Table 3), except for total phosphorus which was significantly lower in the Si-deprived group (105 ± 2 mg P/g vs. 117± 3 mg P/g in the Si-supplemented group; *P* < 0.001; Table 3) even when allowing for multiple testing. Thus the Ca:P ratio in the Si-deprived group was 1.84:1 compared to 1.62:1 in the Si-supplemented group (*P* = 0.0003). Both tibia phosphorus and tibia Ca:P ratio correlated with fasting serum Si concentrations at necropsy (*r* = 0.45, *P* = 0.022 and *r* = − 0.42, *P* = 0.031). Body weights did not differ between the two groups throughout the study period (339 ± 41 g in the Si-deprived vs. 338 ±39 g in the Si-supplemented group at the end of the study, at 26 wk; Fig. 2), but body lengths were *greater* for the *Si-deprived* animals from week 18 until the end of the study (*P* ≤ 0.046 → *P* < 0.01; 45 ± 1 cm vs. 43 ±2 cm in the Si-supplemented group at the end of the study, at 26 wk). Moreover, at necropsy, this difference was reflected in all bone lengths studied (femur, tibia and humerus), with *longer* bones in the *Si-deprived* animals (3.80 ± 0.08 cm, 4.04 ± 0.07 cm and 2.91 ± 0.06 cm, respectively for the femur, tibia and humeri in the Si-deprived group vs. 3.67 ±0.08 cm, 3.93 ± 0.01 cm and 2.82 ± 0.07 cm in the Si-supplemented group; Fig. 3; all *P* ≤ 0.002), again significantly so even when allowing for multiple testing. Interestingly, both body length and bone lengths correlated *inversely* with fasting serum Si concentrations at necropsy (body length: *r* = − 0.62 and *P* = 0.006; bone lengths: all *r* = − 0.5 and *P* < 0.02, Fig. 3). Examination of the growth plates indicated a decrease in growth plate thickness, while chondrocyte density appeared to be increased in the Si-deprived animals compared to the supplemented group although this was not formally quantified (Fig. 4).

**Fig. 5:**
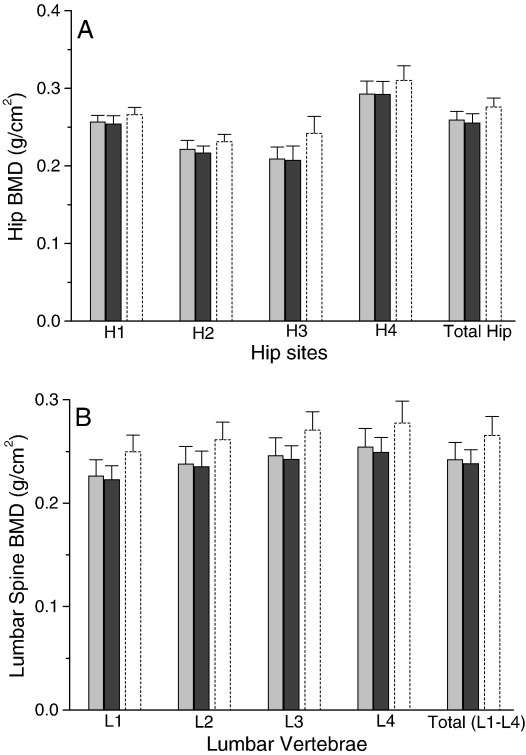
Bone mineral density (BMD) at the hip (four hip sites and total hip; A) and lumbar spine (L1–L4; B) of rats in the Si-deprived (grey bars; *n* = 20 rats) and Si-supplemented (black bars; *n* = 10 rats) groups. There were no significant differences between the two groups (Si-deprived vs. Si-supplemented; unpaired two-tailed Student's *t*-test). BMDs are also shown for the standard rodent stock feed-fed group as a reference for normal rats (hollow dash bars).

**Fig. 6:**
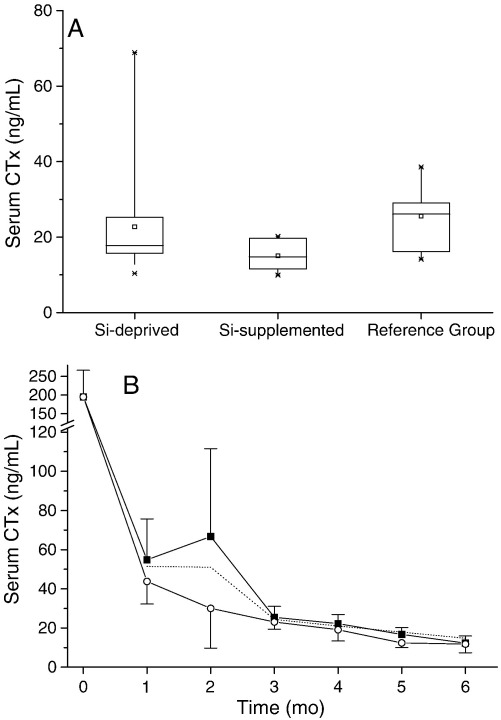
Serum CTx measured at necropsy (A) in the Si-deprived (*n* = 20 rats), Si-supplemented (*n* = 10) and standard rodent stock feed-fed reference (*n* = 10) groups. Serum CTx was significantly lower (*P* = 0.04; unpaired two-tailed Student's *t*-test) in the Si-supplemented group compared to the Si-deprived group. However, there was no significant difference between the two groups (Si-deprived (squares) and Si-supplemented (circles)) in the monthly samples collected during the study (B). Mean monthly serum CTx is also shown for the standard rodent stock feed-fed group as a reference for normal rats (dotted line).

However, in spite of the above differences, extensive measures of bone quality (BMD, bone markers, mechanical testing and histomorphometric analyses) revealed no differences between the groups at necropsy (Fig. 5 and Tables 4 and 5). The only possible difference at necropsy was for serum CTx (Fig. 6A), being apparently lower in the Si-supplemented group (15 ± 4 ng/mL vs. 23 ± 13 ng/mL in the Si-depleted group; *P* = 0.04), but when allowing for multiple testing (i.e. multiple comparisons of bone markers) or when following the monthly changes in CTx over the course of the study from tail vein sampling (Fig. 6B), this difference did not persist. Finally, when examining the values (means ± SD) for some measures of bone quality in the standard rodent stock feed-fed reference group, it was apparent that, without formal analyses, some would differ markedly from those of animals receiving the formulated diet (e.g., iPTH, bone formation rate, trabecular separation and BMD).

## Discussion and conclusions

Our findings illustrate several important issues.

(1) Selective Si deprivation led to a minor drop in serum Si concentrations but a marked fall in urinary Si output (Fig. 1). The current paradigm is that orthosilicic acid [Si(OH)_4_] – a small soluble, labile (i.e. weak or negligible interactions with proteins and other biomolecules) and neutral species – follows the water pool and thus, following absorption, is excreted in line with renal filtration [4,48,49] without either active excretion or retention. In the reference group of standard rodent stock feed-fed animals the ratio of urinary Si (creatinine corrected):serum Si concentrations was 11 ± 6, and similar to that of the Si-supplemented group of 20 ± 14. However, in the Si-deprived animals the ratio was 3 ± 3. This suggests that, in states of Si deprivation, urinary Si conservation, perhaps through renal reabsorption, *can* occur. The kidneys from these two groups of rats are being investigated by gene array and subtractive hybridisation, aiming to identify the first mammalian Si-responsive genes/silicate transporters. Silicon transporters have been identified in diatoms [50] and in the rice plant [51–53], but non so far in mammals. In addition we are investigating differences in aquaporin (AQP) expression in the kidney RNA samples, as the rice Si transporter (Lsi1; [52,53]) shows some similarities (homology) with mammalian kidney AQPs. One or more AQPs may be differentially expressed in the kidneys of the Si-deprived group, suggesting potential Si transport (i.e. the reabsorption of Si from urine), explaining our finding of urinary Si conservation in this group. It may also provide the basis of a test for the assessment of Si status.
(2) The finding that Si supplementation of drinking water did not change bone Si concentrations is instructive. Despite increasing dietary Si intakes by 24-fold throughout the study, there was no incorporation into bone above that achieved in the baseline Si-deprived group (Fig. 1C), and concentrations were five fold lower than those of the standard rodent stock feed-fed reference group. Comparison of urinary concentrations indicated that Si uptake into the circulation was effective in the Si-supplemented group and comparable to that of the standard rodent stock feed-fed reference group (Fig. 1B). Thus, the lack of incorporation into bone (Fig. 1C) was not a failure of absorption but, rather, one of utilisation. This may suggest that some co-factor, probably nutritional, is required for maximal Si uptake into bone and that this co-factor was absent for animals on the formulated low-Si feed. Indeed, the significantly reduced (or sub-optimal) body weight and BMD of rats on the formulated feed, compared to standard rodent stock feed-fed rats (Fig. 2), does suggest that the different nutritional contents of these feeds translate to physiological effects. By considering the differences between these feeds it may be possible to identify dietary component(s) that facilitate Si entry into bone. Major differences in the two diets were in vitamins K, B2, B3, B5 and minerals including cobalt, magnesium, iron and zinc. Vitamin K, although adequate in the diets, was 15-times lower in the formulated low-Si feed. Vitamin K is important for bone health as a co-factor for the carboxylation of osteocalcin, the most abundant non-collagenous protein in bone [54,55]. Osteocalcin is involved in bone mineralisation, but exact mechanisms are not known. Vitamin K deficiency results in an increase in undercarboxylated osteocalcin and increased bone loss, low bone mineral density and increased fractures [54]. van Summersen [55], recently reported that a better vitamin K status was associated with more pronounced increase in BMD in young children. Thus there is suggestion that vitamin K status may influence bone mineralisation and BMD, which could influence the incorporation of minerals including Si into bone. Serum osteocalcin, but not undercarboxylated osteocalcin, was measured in the three groups and although no difference was found between the groups, undercarboxylated osteocalcin is the marker of vitamin K status [54,55]. Interestingly, however, fasting serum and bone (tibia) elements were not different (except for Si) in the rats on the two diets, suggesting the missing co-factor was specific to Si incorporation and not to other elements/minerals.
(3) Despite Si supplementation having no effect on bone Si concentrations or on multiple markers of bone quality when using the specially formulated low-Si feed, we did observe effects on certain other outcomes. Even with robust adjustment for multiple comparisons where appropriate, there were clear differences in (a) the phosphorus content of bone (b) the length of the animals from week 18 onwards, and (c) the length of the bones at necropsy, with an apparent reduction in growth plate thickness and an increase in chondrocyte density. These data may indicate that while circulating Si can influence chondrocyte function directly (c.f. correlations between bone lengths and serum Si; Fig. 3), effects on osteoblast function and/or bone quality may require the incorporation of Si into bone.

Previous studies have reported that Si supplementation marginally increases Ca and P in calves [21] and in the tibia of quails supplemented with Si [40,56]. The increased serum P in the Si-supplemented rats, compared to Si-depleted rats, suggests that Si increases the availability and utilisation (incorporation into bone) of dietary P [40]. Feed intake in Si-supplemented rats was lower than Si-depleted rats (49.2 ± 2.0 g/kg body weight versus 52.6 ± 1.8 g/kg body; *P* = 0.015); water intake was not different 73.7 ± 6.1 g/kg body weight versus 72.5 ± 2.6 g/kg body weight (*P* = 0.75).

**Fig. 7:**
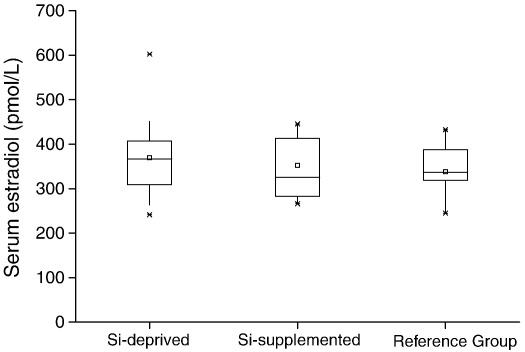
Serum estradiol measured at necropsy in the Si-deprived (*n* = 20 rats), Si-supplemented (*n* = 10) and standard rodent stock feed-fed reference (*n* = 10) groups. There was no significant difference between the two groups (Si-deprived vs. Si-supplemented; unpaired two-tailed Student's *t*-test), nor was there any association with the other measures collected (serum Si, bone length, body length, etc.).

Interestingly the effects that we observed at the chondrocyte-dominated growth plate have resonances with estrogen-deficient states. Estrogen deficiency in men, either through aromatase deficiency [57] or estrogen receptor defects [58], leads to a failure to close the growth plate and accelerated longitudinal and radial bone growth leading to longer bones. In rats, however, the growth plate never closes so the effects of estrogen are more difficult to determine although estrogen clearly plays a role in longitudinal growth. In the current study the thinner growth plate in the Si-depleted rats may be similar to the effect of estrogen. Although at necropsy total serum estradiol concentrations were not different between the two groups (Fig. 7) nor was there any association between serum estradiol and bone length, body length, serum Si or the other measures collected (data not shown). It may be that the expression of the estrogen receptors would have been a more appropriate measure [59,60], but this was not assessed here. Radial measurement of the femurs and serum IGF-1 tended to be higher in the Si-deprived rats, but this did not reach statistical significance (*P* = 0.28 and *P* = 0.09, respectively; data not shown). Moreover, we have some evidence for a Si-estradiol interaction both *in vitro* (Jugdaohsingh and Powell unpublished observations) and *in vivo* where epidemiological studies have shown a positive association between dietary Si intake and bone mineral density; strongly so in pre-menopausal women, less so in men and not at all in post-menopausal women [18]. These results, in pre- and post-menopausal were recently replicated in a UK (women-only) cohort [16]. We showed a strong association in pre-menopausal women, a decrease in the association when peri-menopausal women were included and no association in post-menopausal women. In addition we showed that the positive association (correlation) between Si intake and BMD is restored in post-menopausal women taking hormone replacement therapy [16]. Associations were strongest in current HRT users and slightly less, but still significant, in past users of HRT. Whether Si is required for the effective bone activity of estradiol (or *vice versa*) and how it modifies growth plate dynamics (cell proliferation, cell hypertrophy) should be addressed in further studies. This study is the first to report this *in vivo* effect of Si (i.e. on longitudinal growth), despite apparent severe Si deficiency in the original studies by Carlisle [10] and by Schwarz and Milne [11]. However in these earlier investigations immature male Leghorn cockerels [10] or male Fisher 344 rats [11] were used and, more importantly, the studies were short term (3–5 weeks); our data suggest that such differences in bone length and thus body lengths would begin at puberty (~ 12 weeks in the rat; Fig. 3). Although, female rats were used here, we hypothesis that similar results would have been obtained in male rats, although the magnitude of the differences may have be less based on our epidemiological findings [18].

In conclusion, as with many studies since the 1970s, we were unable to reproduce the profound Si deficiency state reported in rats by Schwarz and Milne [11], and in chickens by Carlisle [10]. We presume that either our dietary Si levels were still too high in the Si-deprived formulated diet, or that these older studies were observing some co-deficiency with their diets, or that our diet produced some co-deficiency that did not allow Si incorporation into bone. It is also possible that our animals were pre-loaded with Si perinatally, prior to transfer to the low-Si diet at three weeks of age. Indeed, Schwarz and Milne [11] removed the normal chow pellets from the nursing mothers (17th day post-partum) to minimise Si exposure to the pups. Carlisle [10] used chicks so that the low-Si diet could be fed at a very early age and, additionally, their yolk sacs were removed (i.e. the chicks were deutectomised), again to minimise maternal Si transfer. In further work, the use of second-generation offspring from Si-deprived mothers may help to reduce body stores of Si.

We cannot explain why the supplemental Si failed to replete bone Si levels to those observed in the reference group of animals on a standard rodent stock diet or, indeed, at least beyond those observed in the Si-deprived group (Fig. 1). Apart from the possible absence of an essential dietary co-factor as noted above, it is also possible that our Si-supplemented group received too little Si to detect a difference compared to the Si-deprived animals. Thus Schwarz and Milne [11] supplemented their rats with 500 µg Si/g diet and noted that lower levels of supplementation gave lesser and statistically insignificant results: they wrote, “… it seems that relative to other trace elements, the amount of Si needed to be quite large ….” [11]. Carlisle [10] supplemented the chick diet with 100 µg Si/g diet, which is a similar level to that of standard rodent stock feed. Our rats received 53 µg Si/g in their drinking water.

However, in the last twenty years, some, but not all, studies have at least reported quantitative changes to bone markers in Si-deprivation animals, if not profound changes in bone and connective tissue health (reviewed by Sripanyakorn et al. [48]). No consistent pattern has emerged to suggest that any one marker or pattern of markers is always influenced by Si status, although markers of collagen turnover do tend to be the most often affected [48]. As we did not show a reduction in bone Si in the deprived group, a corresponding reduction on bone quality, including markers of collagen turnover, is unsurprising. Nonetheless, the observations on urinary conservation of Si and changes to bone/body length, growth plate and bone-phosphorus concentrations are significant, novel and deserve further study. These findings, as well as the hypothesis that some co-factor is required for efficient Si uptake into bone, may throw light on the elusive biological role of Si.

## Figures and Tables

**Table 1:** Compositions of the formulated low-silicon and standard rodent stock diets

Components	Formulated low-Si diet		Standard rodent diet^a^	
	g/kg	% total diet (w/w)	g/kg	% total diet (w/w)
Protein	1">120^b^	12		18
Carbohydrate and fibre	2">400^c^	40		61
Glucose	4.38	0.44		
Sucrose	367	36.7		
Oil	3">50^d^	5		2.5
Choline	0.56	0.056	1.22	
l-Methionine	10	1		0.3
l-Lysine	5	0.5		1
Cystine				0.57
Ash				5.5
Vitamin mix	4.55 g/kg	0.455% TD		
Mineral mix	20	2		4.40

Vitamin mix composition	mg/kg	IU/kg	mg/kg	IU/kg

dl-A-trocophenol (vit. E)	200			104
Vitamin A	16	8000		12000
Vitamin D3	3.8	1520		1500
Menadione (vit. K)	1		15	
Biotin (vit. B7)	1		0.334	
Folic acid (vit. M)	2		2.5	
Inositol	50			
Niacin (vit. B3)	30		78	
D-Ca pantothenate (vit. B5)	10		26	
Riboflavin (Vit B2)	27		11	
Thiamine HCl (vit. B1)	10		14	
Pyridoxine HCl (vit. B6)	15		14	
Cyanocobalamin (vit. B12)	0.05		0.02	
PABA (vit. Bx)	5			

Mineral mix elemental composition	Reported mg/kg	Observed^e^ mg/kg (SD)	Reported mg/kg	Observed^e^ mg/kg (SD)

Ca	5006	5387 (367)	8800	8849 (1481)
K	3659	3271 (725)	7000	7714 (476)
Na	828		3400	
Mg	499	412 (16)	1600	1680 (81)
Mn	50.4	55.6 (10.9)	68	66.1 (4.0)
Zn	44.7	49.7 (6.7)	95	103.8 (18.6)
Fe	35.2	36.2 (1.3)	104	145.4 (10.6)
Cu	5.09	4.57 (1.03)	20	18.3 (1.98)
Se	0.15		0.36	
Mo	0.25			
B	1.05	0.98 (0.17)		7.42 (0.62)
Co	0.002		0.46	
Si	N/P	3.19 (0.64)	N/P	322.4 (47.3)
P	2276		7000	
F	0.91			

a 5consists of wheat meal, barley meal, Soya meal, wheat feed, fish meal, fats and oils, minerals and trace elements, molasses, N/P = not provided, TD = total diet. Consists of wheat meal, barley meal, Soya meal, wheat-feed, fish meal, fats and oils, minerals and trace elements, molasses.">Consists of wheat meal, barley meal, Soya meal, wheat-feed, fish meal, fats and oils, minerals and trace elements, molasses.

b Washed casein.

c Acid washed corn.

d Corn oil.

e Measured by ICPOES following microwave digestion.

**Table 2:** Fasting serum elements in the Si-deprived, Si-supplemented and reference group of animals

	Groups		
Serum Concentrations	Si-deprived (*n* = 20)	Si-supplemented (*n* = 10)	Reference group (*n* = 10)
Ca (mg/L)	149 (3.4)	149 (3.5)	146 (4.6)
P (mg/L)	242 (19)	295 (33)	275 (40)
Mg (mg/L)	43.8 (1.2)	44.1 (0.59)	46.8 (1.9)
K (mg/L)	452 (28)	482 (25)	491 (37)
Zn (mg/L)	1.17 (0.03)	1.21 (0.04)	1.09 (0.07)
Cu (mg/L)	2.05 (0.09)	1.88 (0.10)	2.04 (0.10)
Mn (μg/L)	11.8 (4.3)	14.2 (7.6)	12.2 (3.7)

Means (± SE) of samples collected at necropsy. No significant differences between the Si-deprived and Si-supplemented groups (Student *t*-test). Reference group is standard rodent stock feed-fed animals.

**Table 3:** Element concentrations in tibia in the Si-deprived, Si-supplemented and reference group of animals

Element	Groups		
	Si-deprived (*n* = 20)	Si-supplemented (*n* = 10)	Reference group (*n* = 10)
Ca (mg/g)	193 (1)	190 (2)	192 (1)
P (mg/g)	105 (2)	117 (3)⁎	113 (3)
Na (mg/g)	5.07 (0.11)	5.21 (0.05)	5.16 (0.07)
Mg (mg/g)	3.29 (0.04)	3.29 (0.05)	3.63 (0.03)
K (mg/g)	1.33 (0.04)	1.39 (0.03)	1.19 (0.03)
Zn (mg/g)	0.227 (0.002)	0.228 (0.006)	0.185 (0.003)
Fe (mg/g)	0.076 (0.006)	0.083 (0.012)	0.069 (0.006)
Cu (μg/g)	1.06 (0.06)	1.05 (0.12)	0.875 (0.037)
Mn (μg/g)	0.214 (0.008)	0.196 (0.014)	0.186 (0.007)

Means (± SE). ⁎*P* < 0.001 versus Si-deprived group (Student's *t*-test). No other significant differences between the Si-deprived and Si-supplemented groups. Reference group is standard rodent stock feed-fed animals.

**Table 4:** Bone remodelling markers in the Si-deprived, Si-supplemented and reference group of animals

Markers	Groups		
	Si-deprived (*n* = 20)	Si-supplemented (*n* = 10)	Reference group (*n* = 10)
*Serum*			
Osteocalcin (ng/mL)	129 (13)	140 (20)	123 (17)
ALP (U/mL)	1.36 (0.09)	1.35 (0.07)	1.38 (0.13)
Osteopontin (ng/mL)	29.6 (2.3)	32.2 (2.6)	33.3 (2.7)
Sialic acid (mg/100 mL)	144 (4)	136 (5)	138 (4)
iPTH (pg/mL)	218 (35)	174 (30)	433 (71)
TRAP (U/L)	3.27 (0.41)	3.18 (0.40)	3.62 (0.45)
PYD (nmol/L)	4.12 (0.60)	4.04 (0.54)	3.45 (0.39)

*Urine*⁎			
CTx (μg/mmol cr)	0.619 (0.162)	0.392 (0.163)	0.827 (0.282)
Helical peptide (μg/mmol cr)	30.9 (3.0)	27.2 (1.7)	45.0 (2.4)
PYD (nmol/mmol cr)	21.5 (1.5)	22.9 (2.6)	23.9 (1.7)
Total PYD (nmol/mmol cr)	453 (106)	350 (27)	463 (58)
DPD (nmol/mmol cr)	41.4 (7.7)	46.1 (6.0)	21.5 (5.6)

*Femur* hydroxyproline (mg/g bone)	22.4 (0.4)	21.9 (0.4)	20.6 (0.3)

Means (± SE). ⁎Urine samples were only available for eight rats in the Si-deprived group, five rats in the Si-supplemented group and seven rats in the reference group. ALP = alkaline phosphatase, iPTH = intact parathyroid hormone, TRAP = tartrate resistant acid phosphatases, PYD = pyridinium cross-link, DPD = deoxy-pyridinium cross-link, CTx = C-terminal telopeptide of type I collagen. No significant differences between the Si-deprived and Si-supplemented groups. Reference group is standard rodent stock feed-fed animals.

**Table 5:** Mechanical and histomorphometric analyses of the femur and tibia from the Si-deprived, Si-supplemented and reference group of animals

	Groups		
	Si-deprived (*n* = 20)	Si-supplemented (*n* = 10)	Reference group (*n* = 10)
*Fracture parameters (femur)*			
Torsion moment (Nm)	0.67 (0.09)	0.67 (0.07)	0.74 (0.10
Torsion angle (°)	10.6 (1.9)	11.1 (0.8)	11.3 (1.9)
Torsion stiffness (Nm/rad)	7.2 (1.7)	6.5 (0.5)	7.6 (1.9)
Energy absorbed (J)	0.073 (0.014)	0.076 (0.010)	0.086 (0.020)

*Histomorphometric (tibia)*			
Bone area (%)	25.9 (6.6)	24.2 (11.1)	33.7 (6.2)
Osteoid area (%)	0.73 (0.83)	0.49 (0.48)	0.24 (0.21)
Osteoid width (μm)	2.93 (0.51)	2.81 (0.46)	2.76 (0.21)
Osteoblast perimeter (%)	35.7 (24.3)	19.6 (16.8)	19.2 (21.8)
Osteoclast perimeter (%)	16.4 (17.4)	13.7 (11.7)	12.3 (9.0)
Mineral apposition rate (μm/day)	2.19 (0.38)	2.33 (0.46)	2.15 (0.69)
Bone formation rate (μm^2^/mm^2^/day)	875 (617)	912 (883)	554 (355)
Trabecular thickness (μm)	60.3 (9.8)	65.6 (10.0)	66.2 (10.4)
Trabecular number (mm-1)	5.47 (1.13)	4.54 (1.64)	6.47 (0.62)
Trabecular separation (μm)	135 (66)	202 (167)	89.7 (20.4)

Means (± SE). No significant difference between the Si-deprived and Si-supplemented groups. Reference group is standard rodent stock feed-fed animals.
